# Overt Hyperthyroidism in Third Trimester of Pregnancy: A Case Report

**DOI:** 10.31729/jnma.5062

**Published:** 2020-08-31

**Authors:** Madan Khadka, Achala Thakur, Dipti Das, Akshat Mishra

**Affiliations:** 1 Department of Obstetrics and Gynecology, B.P. Koirala of Institute of health sciences, Dharan, Nepal; 2 Department of Internal Medicine, B.P. Koirala of Institute of health sciences, Dharan, Nepal

**Keywords:** *hyperthyroidism*, *pregnancy*, *PPROM*, *thyrotoxicosis*

## Abstract

Hyperthyroidism is a state of excessive thyroid function. The most common cause of hyperthyroidism is Graves' disease. Overt hyperthyroidism if not treated can have serious outcome on the mother and the fetus. We present a pregnant women at 31 weeks of gestation presented with shortness of breath and palpitation with previous history of caesarean section and was treated with propyl thiouracil, beta blockers, antihypertensive drug, and during her course of treatment had Preterm Prelabor Rupture of Membrane with subsequent onset of labor and had normal vaginal delivery of 1.7 kg healthy baby. This report emphasize on the timely management of overt symptoms before the onset of labor.

## INTRODUCTION

The incidence of hyperthyroidism in pregnancy varies between 2-17 per 1000 live birth.^1^ Graves' disease accounts for 60-80% of hyperthyroidism.^2^ Pregnant mother if untreated can present as wide varied symptoms depending upon severity of thyrotoxicosis. Serious symptoms include supra ventricular tachycardia, atrial fibrillation, thyroid storm, compression of optic nerve leading to papilledema and thus permanent loss of vision. ^2^ Inadequate treatment of maternal hyperthyroidism is associated with prematurity, low birth weight, and stillbirths.^3^

## CASE REPORT

A 26 years old G_2_P_1_L_1_ at 31 weeks of pregnancy complaints of shortness of breath and palpitation since 4-5 months and raised blood pressure since 24 weeks of gestation. She gives history of neck swelling since last 2 years and had taken some thyroid medication for 6-7 months but left medication 4 months back on her own. She had history of previous caesarean section done for non progress of labor 6 years back.

On examination patient was dyspnoeic, respiratory rate was 22/min, pulse 130/min, BP 140/80, with saturation of 88% at room air. Systemic examination revealed exophthalmos and diffuse thyroid swelling of 3*3cm was seen. Respiratory examination revealed normal vesicular breath sounds. Systolic murmurs were heard at aortic and mitral area and per abdominal examination revealed uterus corresponding to 28-30 weeks, with single live intrauterine fetus in longitudinal lie not in labour without scar tenderness.

On Investigation, her complete blood count, renal function test, LDH, serum uric acid, cardiac markers and LFT were normal. Her thyroid function was grossly deranged. fT3, fT4 and TSH were 37 pg/ml, 106 pg/ ml and 0.001 uIU/ml respectively. Maternal TRAb couldn't be sent due to unavailability of facility. ECHO suggestive of mild MR, moderate TR, moderate PAH, LVEF 60-65%, <50% collapsibility of left ventricle with inspiration.

She was started with Propyl thiouracil 100 mg TDS; Labetalol 200 mg TDS; Hydrochlorothiazide 12.5 mg. OD; Propranolol 20 mg BD, and Iron and Calcium tablets. Two doses of dexamethasone were given for fetal lung maturity. Patient had significant improvement in term of shortness of breath and palpitation after initiation of treatment.

During her 5^th^ day of course of treatment, she had Preterm Prelabor Rupture of Membrane (PPROM), and IV antibiotics was started to prevent maternal and fetal infection. On subsequent day, she progressed into labor. The patient was counselled regarding the mode of delivery and its possible risk. VBAC (vaginal delivery after caesarean) was counselled with short 2^nd^ stage of labor to be aided by instrumentation. Caesarean section was deferred due to possible intra operative and postoperative complication like supra ventricular tachycardia, atrial fibrillation and thyroid storm.^4^ With careful monitoring, patient delivered a healthy female baby weighing 1.705 kg vaginally without instrumentation.

The baby was evaluated by neonatologist. Baby was then handed over to the mother. Her TFT was sent after 72 hrs. Mother was evaluated during postpartum period. Antithyroid drug was continued and she was discharged on 4^th^ postpartum day with advice to follow up in Medicine OPD. Patient is currently on antithyroid medication and is asymptomatic and regularly on follow up in medicine OPD.

## DISCUSSION

Thyrotoxicosis is the most frequent thyroid disorder in the pregnant patient and is the most difficult to evaluate and manage. Dealing with hyperthyroidism as a cause of thyrotoxicosis is difficult since radioactive iodine is contraindicated in pregnancy.^3^ Most common cause of hyperthyroidism is grave disease. Other important common causes are toxic nodular goitre, thyroiditis, iodine induced, drug induced or factitious ingestion of excess thyroid hormones.^2^ Inadequate treatment of maternal hyperthyroidism is associated with prematurity, low birth weight, and stillbirths.^3^ Hyperthyroidism clinically present as hyperactivity, irritability, heat intolerance, sweating, weight loss with increased appetite, tachycardia, goitre, lid lag, muscle weakness.^2^

Patient presented with shortness of breath and palpitation with neck swelling, excessive sweating and history of drugs intake for thyroid disease suggestive of thyroid disorder, however differential diagnosis were underlying heart disease, complication of pregnancy induced hypertension. After careful evaluation, we concluded the diagnosis of pregnancy with overt hyperthyroidism with pregnancy induced hypertension with valvular heart disease with previous caesarean section.

Management required intensive monitoring and supportive care, identification and treatment of precipitating cause, and measures to reduce the fetal complication. American Thyroid Association and American Association of Clinical Endocrinologist (2011) recommend Methimazole as a first choice for non pregnant and Propyl Thiouracil (PTU) to be recommended in first trimester followed by Methimazole beginning in the second trimester.^5^ PTU has been the preferred thionamide in United States.^6^ It can be started as 50-150 mg orally three times a day depending upon severity.^5^ PTU has been associated with Hepatotoxicity. Methimazole has been associated with a rare embryopathy that includes esophageal or choanal atresia and aplasia cutis. We started propyl thiouracil 100 mg three times a day as antithyroid medication. Radioiodine treatment is absolute contraindication in pregnancy and breast feeding mother. Indication for surgery like subtotal or near total thyroidectomy are reserved for patient who are refractory to medical management or who cannot adhere to medical treatment or in whom the drug therapy proves toxic.^7^

Blood pressure was optimized with labetalol 200 mg three times a day with diuretics hydrochlorothiazide 12.5 mg OD and beta blocker propranolol 20 mg twice a day to reduce the tachycardia and prevent high output cardiac failure.

The patient had PPROM on the 5^th^ day of admission which was managed with prophylactic IV antibiotics (ampicillin and metronidazole) to prevent maternal and fetal complication. Monitoring of leaking patient is complicated by onset of labor that confuses treating clinician from subclinical chorioamnionitis with hyperthyroidism and scar dehiscence in a patient with pregnancy caesarean section as tachycardia predominates in all above conditions. With careful monitoring throughout labor, the patient delivered vaginally, a 1.7 kg healthy baby.

We concluded that an overt symptoms of hyperthyroidism needs urgent and timely management to prevent deleterious effect on mother and the fetus.

## Consent:

**JNMA Case Report Consent Form** was signed by the patient and the original article is attached with the patient's chart.

## Conflict of Interest

**None.**
